# Is it beneficial to add laser ablation to curettage in the treatment of pilonidal sinus disease?

**DOI:** 10.1007/s10103-025-04409-8

**Published:** 2025-03-18

**Authors:** Mehmet Ali Demir, Tahsin Çolak, Cumhur Özcan, Hüseyin Oğuzhan İnan, Erkan Güler

**Affiliations:** Mersin University School of Medicine, Mersin, Turkey

**Keywords:** Pilonidal sinus, Laser therapy, Curettage, Postoperative complications, Wound healing, Recurrence

## Abstract

**Aim:**

This study aimed to compare laser application with sinus curettage against only sinus curettage in the treatment of pilonidal sinus disease (PSD) in terms of postoperative complications, wound healing, and recurrence. Additionally, we aimed to investigate factors associated with wound healing and recurrence after PSD surgery.

**Patients and methods:**

This study included patients diagnosed with PSD between February 2019, and September 2022. The patients were randomly assigned to either the laser + curettage (L/C; *n* = 40) group or the curettage-only (C; *n* = 40) group. The following data were collected: demographic and anthropometric information, smoking status, comorbidities, number of PSD-related orifices, complicated or uncomplicated disease, prior recurrence, postoperative findings, wound healing problems within 30 days postoperatively, and recurrence within 6 months postoperatively.

**Results:**

The L/C and C groups were similar in terms of age and sex distribution. The groups had similar results for postoperative pain, discharge, bleeding, wound healing problems at 30 days, and recurrence at 6 months. Multivariable logistic regression revealed that, high weight and higher orifice count were independently associated with wound healing problems. Additionally, high weight, higher orifice count and prior recurrence were independently associated with recurrence.

**Conclusion:**

Combined curettage and laser therapy showed non-significant but broad benefits over curettage alone, but statistical significance was not achieved in any of the adverse findings, as well as wound healing and recurrence. Higher body weight and higher orifice count were associated with both poor wound healing and recurrence, while prior recurrence was another factor associated with recurrence at 6 months.

## Introduction

Pilonidal sinus disease (PSD) is defined as a small cyst or abscess forming at the top of the gluteal cleft that typically contains hair, dirt, debris, and unhealthy granulation tissue [1]. These lesions cause severe pain and often become infected [1, 2]. Surgical management is the primary treatment for particularly chronic PSD and many approaches exist, including curettage, wide excision with primary closure, flap or grafts, and secondary healing. However, none of these methods can be considered as an optimal treatment, and therefore, research is ongoing to identify new approaches or adjunctive surgical options for PSD [2, 3].

For many years, wide excision and secondary intention healing were considered the standard approach for chronic PSD. This method requires prolonged healing and poses significant limitations on normal activities, which led to the development of various reconstructive techniques [3] aiming to remove affected tissue while shifting the scar away from the midline. Despite the reported success of frequently used reconstructive techniques such as Karydakis and Limberg flaps [4], these methods require removal of extensive intergluteal tissue sections, delaying recovery [2]. Over time, minimally invasive methods were developed to address these limitations, such as the endoscopic pilonidal sinus ablation method and the administration of fibrin glue, liquid, crystallized phenol, and thrombin gelatin matrix. These methods are suggested to effectively remove diseased tissue while preserving healthy tissue, and their minimally invasive nature yields favorable short-term outcomes in terms of pain, hospital stay, recovery, and satisfaction [3–6]. However, conflicting outcomes regarding success and recurrence have been reported for these methods when used as monotherapies [3].

Recently, the increasing popularity of laser technology has made laser treatment a promising minimally invasive alternative for PSD treatment, with potential utility as an adjunct to surgical interventions [2, 3]. In the sinus laser therapy approach, hair is removed and the sinus is curetted; then, a radial fiber connected to a diode laser is introduced into the sinus to obliterate the sinus tracts [2, 3]. Although the use of lasers in PSD treatment began in the early 1990s [6], the first report of pilonidal sinus destruction with a radial laser probe was published in 2017 [7]. However, there is a lack of sufficient and high-quality data exploring the short- and long-term outcomes of radial laser treatment for chronic PSD. Therefore, we aimed to compare PSD patients with classical sinus curettage to recipients of adjunctive laser treatment after sinus curettage in terms of post-operative complications, wound healing, and recurrence. The secondary aim was to investigate factors associated with wound healing and recurrence.

## Patients and methods

### Ethical statement

The study commenced after obtaining ethical approval from the Clinical Research Ethics Committee of Mersin. University Faculty of Medicine (date: June 2, 2022, decision no: 2022/04) and written permissions from the Department of General Surgery (reference no: E-79426989-903.99-2120956) for the conduct of the research.

### Patient recruitment and study design

This study included 100 consecutive patients diagnosed with PSD and scheduled for surgery at the General Surgery Department of Mersin University Faculty of Medicine Health Research and Application Hospital, between February 1, 2019, and September 7, 2022. The patients recruited into the study, all over 18 years of age, were willing to participate in the study with at least 6 months postoperative follow-up. They were randomly assigned to either the laser + curettage (L/C; *n* = 50) group or the curettage-only (C; *n* = 50) group. Randomization was performed by an independent statistical consultant after the decision for surgery was made. Patients who declined participation (L/C: 2; C: 1), those using anticoagulants (L/C: 1; C: 1), patients with rheumatic diseases using steroids (C: 1), patients on immunosuppressants (L/C: 1), those under 18 years of age (L/C: 2; C: 1), patients with pilonidal sinus abscess (L/C: 4; C: 5), and those with connective tissue diseases (C: 1) were excluded from the study. In the final analysis, a total of 80 patients, with 40 patients in each group, were included in the study.

### Patient data collection

At the time of admission, preoperative, and follow-up periods, the following data were collected: demographic (age and sex) and anthropometric (height and weight) information, smoking status, comorbidities, PSD-related characteristics (orifices, complicated disease, prior recurrence), postoperative findings (pain, discharge, bleeding), wound healing problems within 30 days postoperatively, and recurrence within 6 months postoperatively. Body mass index (BMI) was calculated by dividing weight (kg) by the square of height (m²). Pilonidal sinuses were categorized into complicated and uncomplicated groups based on the number of orifices identified preoperatively, with patients having 3 or more orifices classified as complicated and those with 2 or fewer as uncomplicated.

### Preoperative period and surgical procedures

Patients with symptomatic PSD were thoroughly evaluated preoperatively through a detailed history of their complaints (pain, discharge, or bleeding), duration of symptoms, comorbid conditions, previous acute episodes, and prior treatments. A comprehensive local examination of the sacrococcygeal region was conducted to assess the number and distribution of pits. The choice of procedure (L/C or C only) was assigned by a statistical consultant except for patients who opposed laser treatment for any reason, after which patients were briefed on the surgical procedure, potential complications, expected recovery rate, postoperative pain, and course. Informed consent was obtained before proceeding with the necessary interventions.

The operations were performed under local anesthesia with patients in the prone position with their hips flexed (Jack-knife position). The direction and length of the sinus tracts were determined by examining the pits, and a diluted hydrogen peroxide and/or saline solution was injected with a small venous catheter to delineate the connections between the pits. Using a punch biopsy (3–5 mm), the overlying skin of the directly associated pits was excised to access the underlying cavities, which were then debrided and cleaned with saline.

In the C only group (only debridement and curettage), the pit openings were excised using a No. 11 scalpel blade, and the pilonidal sinus tract was initially cleaned with a curette. Subsequently, a specially designed brush was used to ensure the complete removal of pseudo-epithelial and debris from all sinus tracts and pouch walls. The tracts were then irrigated with saline. In the L/C group, firstly, the sinus tract(s) were debrided and curetted as mentioned above. Then, a NeoV V1470 diode laser (1470 nm wavelength; Neolaser Ltd, Caesarea, Israel) was used for laser application. The laser probe was inserted through the punch biopsy site to the appropriate depth in the sinus tracts. External compression was applied to collapse the tract around the probe, and the probe was fired in 3-second pulses at 10 watts, repeated 10–30 times for each identified sinus tract. This process was repeated for all sinus tracts, necessitating multiple insertions and firings in many patients.

#### Postoperative care and follow-up

Patients were monitored in the ward for 2–6 h before discharge. Prior to discharge, they were advised to maintain routine hair removal practices from the sacrococcygeal area, shower immediately after each haircut, avoid prolonged sitting, and quit smoking. Patients in both groups were discharged on the same day and prescribed a 1-week course of amoxicillin + clavulanate and non-steroidal anti-inflammatory drugs.

Follow-up visits were scheduled weekly for the first month, once a fortnight in the second month, and at the 3rd and 6th months postoperatively. During follow-up visits, patients were examined by the operating surgeon and a surgical assistant to detect any complications and evaluate wound healing, as well as assess for seroma, hematoma, and surgical site infections. At the 30-day follow-up, wound healing status (complete/incomplete) was recorded, and at the 6-month follow-up, recurrence rates were documented.

### Statistical analysis

All analyses were performed using IBM SPSS Statistics for Windows, Version 25.0 (IBM Corp., Armonk, NY, USA). p-values less than 0.05 were accepted as statistically significant. For the normality check, the Shapiro-Wilk test was used. Descriptive statistics were presented using median (25th percentile − 75th percentile) for non-normally distributed continuous variables and frequency (percentage) for categorical variables. Continuous variables were analyzed with the Mann-Whitney U test due to non-normality of distribution. Categorical variables were analyzed with chi-square tests or the Fisher’s exact test. Logistic regression analyses were performed to determine significant factors independently associated with wound healing and recurrence. To create the multivariable model, variables were first compared between the relevant groups with univariable logistic regression and those with statistical significance were included into the multivariable logistic regression–which was performed via the forward conditional selection method.

## Results

**Fig. 1:**
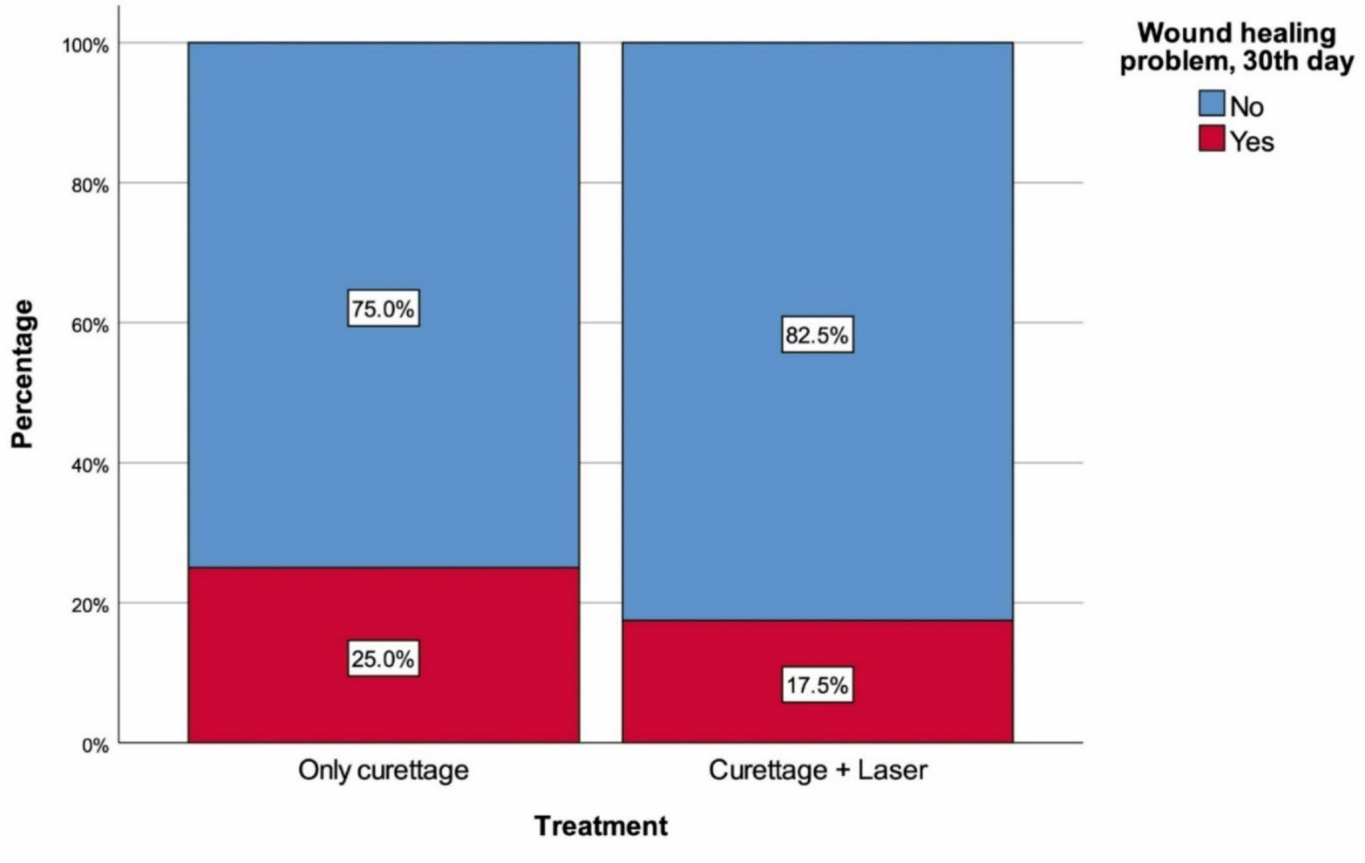
Wound healing problems at 30 days with regard to treatment

**Fig. 2:**
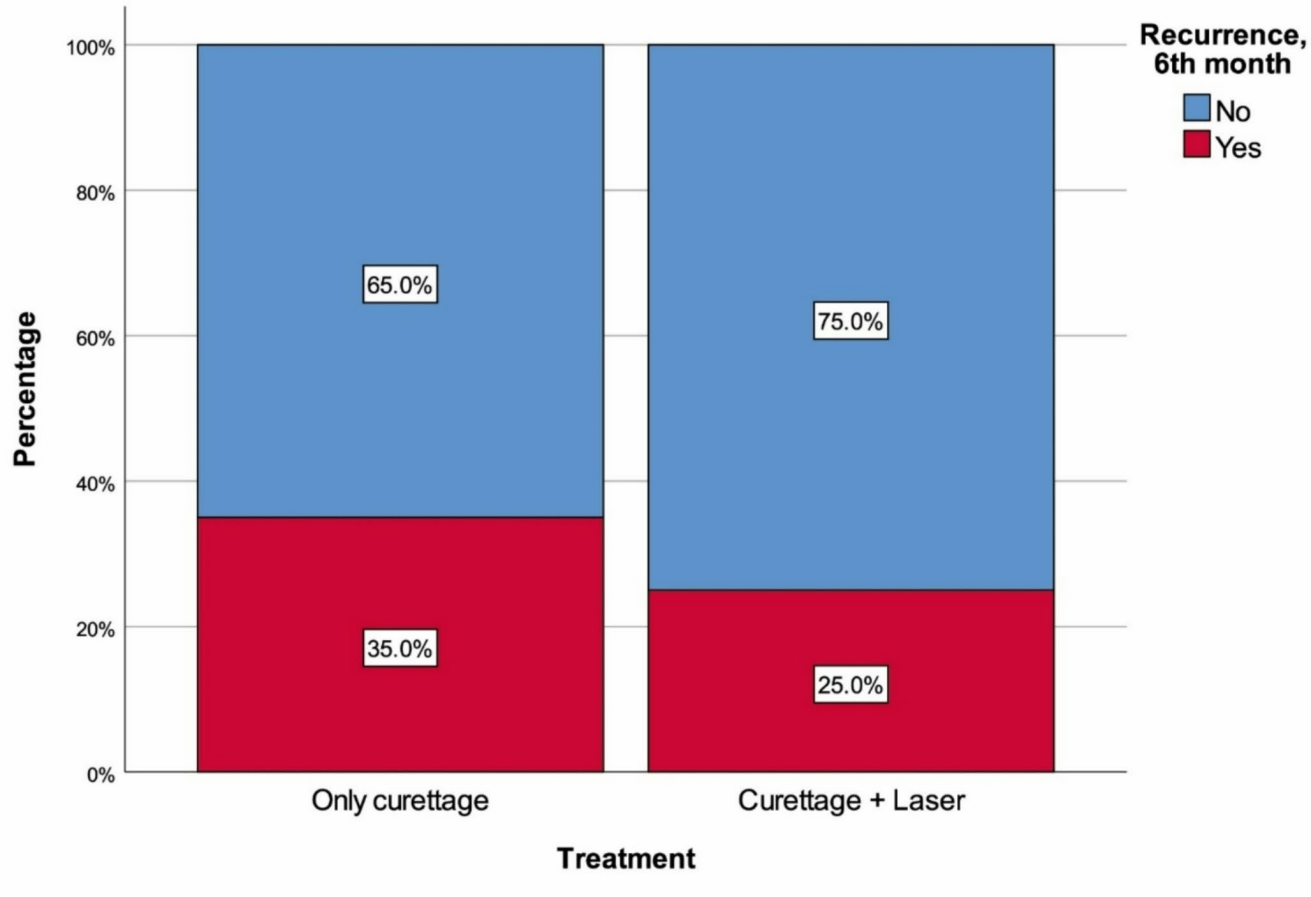
Recurrence at the 6th postoperative month with regard to treatment

The median age of the C group was 23 years (20.5–28), with 85.0% (*n* = 34) being man. The median age of the L/C group was 23 years (20–28), with 85.0% (*n* = 34) being man. There were no significant differences between the groups in terms of age (*p* = 0.923) and sex distribution (*p* = 1.000). Additionally, there were no significant differences between the groups in BMI (*p* = 0.679), smoking (*p* = 0.464), diabetes mellitus (*p* = 1.000), number of orifices detected (*p* = 0.968), frequency of complicated pilonidal sinus (*p* = 0.811), prior recurrence (*p* = 0.348), postoperative pain (*p* = 0.481), discharge (*p* = 0.101), bleeding (*p* = 0.712), frequency of wound healing problems at 30 days (*p* = 0.585), and frequency of recurrence at 6 months (*p* = 0.464) (Table 1; Figs. 1 and 2).

**Table 1:** Summary of variables with regard to treatment group

	Treatment		
	Only curettage (*n* = 40)	Curettage + Laser (*n* = 40)	p
Age, years	23 (20.5–28)	23 (20–28)	0.923^‡^
Sex			
Man	34 (85.0%)	34 (85.0%)	1.000^#^
Women	6 (15.0%)	6 (15.0%)	
Height, cm	175 (171.5–178.5)	177 (171–184)	0.150^‡^
Weight, kg	79 (74–85)	81.5 (71–90)	0.750^‡^
Body mass index, kg/m^2^	25.73 (24.31–28.57)	25.18 (24.18–28.39)	0.679^‡^
Smoking	26 (65.0%)	30 (75.0%)	0.464^#^
Diabetes mellitus	0 (0.0%)	1 (2.5%)	1.000^§^
Number of orifices	2 (1–3)	2 (1–3)	0.968^‡^
Complicated pilonidal sinus	12 (30.0%)	14 (35.0%)	0.811^#^
Prior recurrence	8 (20.0%)	4 (10.0%)	0.348^#^
Postoperative findings			
Pain	6 (15.0%)	3 (7.5%)	0.481^§^
Discharge	18 (45.0%)	10 (25.0%)	0.101^#^
Bleeding	5 (12.5%)	3 (7.5%)	0.712^§^
Wound healing problem, 30th day	10 (25.0%)	7 (17.5%)	0.585^#^
Recurrence, 6th month	14 (35.0%)	10 (25.0%)	0.464^#^

Descriptive statistics were presented using median (25th percentile − 75th percentile) for non-normally distributed continuous variables and frequency (percentage) for categorical variables

‡ Mann Whitney U test, # Chi-square test, § Fisher’s exact test

Next, we examined patients with and without wound healing problems. Multivariable logistic regression revealed that higher weight (OR: 1.084, 95% CI: 1.034–1.136, *p* = 0.001) and higher number of detected orifices (OR: 1.584, 95% CI: 1.099–2.283, *p* = 0.014) were independently associated with the presence of wound healing problems at 30 days (Table 2).

**Table 2:** Odds ratios for wound healing problems, logistic regression analysis results

	Univariable		Multivariable ^(1)^	
	OR (95% CI)	p	OR (95% CI)	p
Age, years	0.962 (0.878–1.054)	0.404		
Sex, Women	0.707 (0.139–3.581)	0.675		
Height, cm	1.111 (1.023–1.208)	**0.013**		0.322
Weight, kg	1.092 (1.042–1.145)	**< 0.001**	1.084 (1.034–1.136)	**0.001**
Body mass index, kg/m^2^	1.428 (1.181–1.726)	**< 0.001**		0.394
Smoking	0.733 (0.236–2.282)	0.592		
Diabetes mellitus	6360932193.726 (0 - N/A)	1.000		
Number of orifices	1.681 (1.219–2.320)	**0.002**	1.584 (1.099–2.283)	**0.014**
Complicated pilonidal sinus	5.867 (1.855–18.553)	**0.003**		0.695
Prior recurrence	3.333 (0.903–12.305)	0.071		
Treatment, Curettage + Laser	0.636 (0.215–1.883)	0.414		
Nagelkerke R^2^	-		0.447	

OR: Odds ratio, CI: Confidence interval, N/A: Non-applicable, (1) Multivariable analysis was performed via forward conditional selection method

The comparison of patients with and without recurrence via multivariable logistic regression showed that higher weight (OR: 1.063, 95% CI: 1.018–1.111, *p* = 0.006), higher number of orifices (OR: 1.587, 95% CI: 1.122–2.245, *p* = 0.009) and prior recurrence (OR: 5.193, 95% CI: 1.149–23.476, *p* = 0.032) were independently associated with recurrence (Table 3).

**Table 3:** Odds ratios for recurrence, logistic regression analysis results

	Univariable		Multivariable ^(1)^	
	OR (95% CI)	p	OR (95% CI)	p
Age, years	0.961 (0.887–1.041)	0.330		
Sex, Women	0.418 (0.084–2.073)	0.286		
Height, cm	1.111 (1.030–1.198)	**0.007**		0.661
Weight, kg	1.079 (1.034–1.126)	**< 0.001**	1.063 (1.018–1.111)	**0.006**
Body mass index, kg/m^2^	1.315 (1.121–1.544)	**0.001**		0.543
Smoking	0.800 (0.286–2.236)	0.670		
Diabetes mellitus	3,933,330,052 (0 - N/A)	1.000		
Number of orifices	1.629 (1.195–2.220)	**0.002**	1.587 (1.122–2.245)	**0.009**
Complicated pilonidal sinus	5.133 (1.828–14.414)	**0.002**		0.777
Prior recurrence	6.500 (1.728–24.444)	**0.006**	5.193 (1.149–23.476)	**0.032**
Treatment, Curettage + Laser	0.619 (0.235–1.627)	0.331		
Nagelkerke R^2^	-		0.432	

OR: Odds ratio, CI: Confidence interval, N/A: Non-applicable, (1) Multivariable analysis was performed via forward conditional selection method

## Discussion

The present study has demonstrated that the treatment of PSD using curettage alone versus curettage combined with laser therapy did not result in significant differences in terms of early postoperative complications, wound healing problems, or recurrence rates. However, notably, the data for wound healing problems (30 days) and recurrence (6 months) show relatively better results with adjunctive laser therapy, which may gain relevance in larger studies that must be performed to obtain more data regarding the utility of laser treatment in PSD. Secondarily, independent risk factors for wound healing issues were identified as high body weight and a high number of pits. For recurrence, independent risk factors included high body weight, a high number of pits, and a history of previous recurrence.

Acute presentations of PSD, such as abscess formation, can often be managed with conservative treatments or simple incision and drainage with high success, while chronic PSD typically requires surgical intervention [2, 3]. Among the numerous surgical techniques described for PSD treatment, none are optimal and current research is focused on generating minimally invasive techniques in order to prevent morbidity and complications, reduce recurrence, and increase patient satisfaction [8]. Laser treatment has been employed in the management of PSD since the early 1990s, with various lasers being used for different purposes. CO_2_ lasers have been utilized for tissue cutting or elliptical excision [7], laser epilation has been applied to reduce PSD recurrence [9], and Nd: YAG (neodymium-doped yttrium aluminum garnet) lasers have been used for both primary and recurrent PSD treatment [10]. Nonetheless, the first report of pilonidal sinus destruction using a radial laser probe dates back only a few years, published a healing rate of 87.5% in 2017 [8]. Although this approach has now been adopted by many, there is a lack of corroborative high-level evidence regarding outcomes, which are necessary to develop evidence-based guidelines for the use of laser treatment in chronic PSD. In this study, we compared simple sinus curettage recipients with patients who had undergone laser application to the sinus after the same curettage procedure. Compared to the curettage-only group, the laser group had significantly fewer adverse outcomes in all examined metrics, including pain (15% vs. 7.5%), discharge (45% vs. 25%), bleeding (12.5% vs. 7.5%), wound healing problems at one month (25% vs. 17.5%), and recurrence within six months (35% vs. 25%). Although the percentages favored the laser treatment group, statistical significance was not achieved for any of the unique comparisons. Nonetheless, pooling of postoperative findings (pain, discharge, and bleeding) would have likely created a significant difference, indicating that adjunct laser therapy could be providing low levels of benefit to several outcomes. Still, the similar results for 30-day wound healing and recurrence demonstrate that adjunctive laser therapy does not appear to improve wound healing or reduce recurrence likelihood.

In a comprehensive systematic review examining the outcomes of laser treatment for chronic PSD, the procedure was deemed a safe and effective option, with a 94% healing rate, a 3.8% recurrence rate, and minimal complications [1], which are outcomes that have been shown to vary moderately in retrospective studies [11, 12] In numerous published studies, laser treatment approaches in PSD surgery have been compared with various treatment protocols. A significant portion of these studies found notable differences, leading to the recommendation of laser treatment approaches due to their advantages. In a prospective randomized study comparing the Karydakis procedure with combined pit excision and laser ablation in patients with uncomplicated early-stage pilonidal disease, laser application was associated with shorter operation time, faster return to normal activities, less pain, and higher patient satisfaction–despite similar recurrence rates [13]. In a recent study, they compared the outcomes of minimally invasive pit excision surgery with and without laser in 221 PSD patients. They reported a significantly lower recurrence rate among laser recipients (8.2% vs. 32.9%). Additionally, their multivariable analysis demonstrated that adding laser to the surgical treatment caused a 4.35-fold reduction in the risk of recurrence [14]. In another recent study, which compared the outcomes of laser ablation with two classic methods, direct closure and flap reconstruction, in 278 PSD cases. Two months postoperatively, the recurrence rates were 3.1% in the laser group, 7.5% in the direct closure group, and 11.7% in the flap group. Complications (infection, hematoma, wound dehiscence) were reported in 12.1%, 26.9%, and 34.6% of patients, respectively [15]. A study examining the benefits of adding laser to endoscopic PSD surgery, found similar wound healing rates between the groups (95.8% with laser vs. 93% without laser). However, they reported that the laser group had significantly shorter surgical, return-to-work, and wound closure times, as well as significantly less pain on postoperative day 14. A notable limitation was that only one recurrence was detected in each group at a median follow-up of 9 months [16].

Although many studies have reported better outcomes with laser treatment, there are also studies that do not report significant results in favor of laser treatment, similar to the present study.In a study, the outcomes of crystallized phenol application were compared with those of laser treatment, and although the postoperative pain was significantly lower in the laser group, no differences were observed between the groups in terms of postoperative recurrence, bleeding, and patient satisfaction [17]. The use of laser treatment in pilonidal sinus surgery has been advocated for several important advantages and disadvantages. The advantages include the simplicity of the method, quick application, accelerated healing, swift return to normal daily activities, minimal postoperative pain, and ability to precisely target and completely remove the sinus tract, which is expected to reduce the likelihood of residual disease and recurrence [2, 3, 18–20]. The disadvantages include high costs, limited availability, difficulties in reaching deep tracts, abscesses, or multiple sites [21–23]. Traditional surgical methods for PSD are associated with relatively high recurrence rates [24, 25]. Adjunctive laser treatment has been associated with lower recurrence rates and better outcomes in a significant portion of studies, despite the presence of a body of literature which has not supported these claims. Nonetheless, when available data is taken together with our findings, it can be suggested that adding laser therapy to curettage treatment has positive effects in reducing postoperative adverse outcomes.

Our results contribute to the growing body of evidence supporting the effectiveness of laser treatment in terms of adverse outcomes following PSD treatment. However, it is important to acknowledge several limitations. First, the study was prospectively-designed with randomized patient allocation, but it was conducted at a single center with a relatively small sample size. Additionally, our study examined recurrence only at the 6-month mark, preventing conclusions about later recurrence events. Moreover, we used a 1470 nm diode laser, so the comparative benefits of different laser wavelengths remain undetermined; however, this limitation is true for the overwhelming majority of literature on this topic. Factors that could influence wound healing, such as connective tissue disorders and the use of medications like steroids, were also not considered in our analysis.

The present data show that the treatment of PSD with curettage combined with laser therapy showed non-significant superiority over curettage alone in terms of early postoperative complications, wound healing issues, and recurrence. Nonetheless, it is safe to state that adjunctive laser therapy reduces the overall likelihood of postoperative adverse findings. Furthermore, we determined that the presence of wound healing problems at 30 days was independently associated with higher body weight and higher pit count. Similarly, high body weight, a high number of pits, and a history of prior recurrence were independently associated with recurrence at 6 months. Further research is needed to clarify the potential benefits of laser treatment in PSD surgery and to better understand the factors influencing recurrence and wound healing following different interventions.

## Data Availability

Data is provided within the manuscript or supplementary information files.

## References

[1] Romic I, Augustin G, Bogdanic B, Bruketa T, Moric T (2022) Laser treatment of pilonidal disease: a systematic review. Lasers Med Sci 37(2):723–732 34291332 10.1007/s10103-021-03379-x

[2] Abdelnaby M, Fathy M, Emile SH, Arnous M, Balata M, Abdelmawla A, Abdallah E (2021) Sinus laser therapy versus sinus Lay open in the management of sacrococcygeal pilonidal disease. Colorectal Dis 23(9):2456–2465 34042233 10.1111/codi.15755

[3] Berthier C, Bérard E, Meresse T, Grolleau JL, Herlin C, Chaput B (2019) A comparison of flap reconstruction vs the laying open technique or excision and direct suture for pilonidal sinus disease: A meta-analysis of randomised studies. Int Wound J 16(5):1119–1135 31230414 10.1111/iwj.13163 PMC7948539

[4] Gavriilidis P, Bota E (2019) Limberg flap versus Karydakis flap for treating pilonidal sinus disease: a systematic review and meta-analysis. Can J Surg 62(2):131–138 30697992 10.1503/cjs.003018 PMC6440894

[5] Tien T, Athem R, Arulampalam T (2018) Outcomes of endoscopic pilonidal sinus treatment (EPSiT): a systematic review. Tech Coloproctol 22(5):325–331 29850944 10.1007/s10151-018-1803-4

[6] Klin B, Heller ON, Kaplan I (1990) The use of the CO2 laser in pilonidal sinus disease: preliminary results of an ambulatory prospective study. J Clin Laser Med Surg 8(1):31 10160877

[7] Dessily M, Charara F, Ralea S, Allé JL (2017) Pilonidal sinus destruction with a radial laser probe: technique and first Belgian experience. Acta Chir Belg 117(3):164–168 28056720 10.1080/00015458.2016.1272285

[8] Ganduboina R, Sreekumar A, Dutta P, Dhawan A, Adhnon A, Soni A et al (2023) Laser ablation: a unique and beneficial therapeutic option for pilonidal sinus? And the potential for further innovation-a review. Lasers Med Sci 38(1):124 37204472 10.1007/s10103-023-03788-0

[9] Doll D, Luedi MM (2017) Laser May reduce recurrence rate in pilonidal sinus disease by reducing captured occipital hair. Lasers Med Sci 32(2):481–482 27549332 10.1007/s10103-016-2053-1

[10] Brehmer F, Zutt M, Lockmann A, Schön MP, Thoms KM (2013) Nd:YAG laser epilation to prevent recurrences after pilonidal sinus surgery. J Dtsch Dermatol Ges 11(12):1203–1205 24267020 10.1111/ddg.12227

[11] Bonito F, Cerejeira D, Goulão J, de Assunção Gonçalves J (2021) A retrospective study of the safety and efficacy of a radial diode laser probe in the management of pilonidal sinus disease. Dermatol Surg 47(9):1224–1228 33988547 10.1097/DSS.0000000000003080

[12] Li Z, Jin L, Gong T, Qin K, Cui C, Wang Z, Wu J (2023) An effective and considerable treatment of pilonidal sinus disease by laser ablation. Lasers Med Sci 38(1):82 36856904 10.1007/s10103-023-03741-1 PMC9977879

[13] Yardimci VH (2020) Outcomes of two treatments for uncomplicated pilonidal sinus disease: Karydakis flap procedure and sinus tract ablation procedure using a 1,470 Nm diode laser combined with pit excision. Lasers Surg Med 52(9):848–854 32064640 10.1002/lsm.23224

[14] Horesh N, Maman R, Zager Y, Anteby R, Weksler Y, Carter D et al (2023) Surgical outcomes of minimally invasive Trephine surgery for pilonidal sinus disease with and without laser therapy: a comparative study. Tech Coloproctol 28(1):13 38093161 10.1007/s10151-023-02897-w

[15] Tyrväinen E, Nuutinen H, Savikkomaa E, Myllykangas HM (2024) Comparison of laser ablation, simple excision, and flap reconstruction in the treatment of pilonidal sinus disease. Lasers Med Sci 39(1):52 38291247 10.1007/s10103-024-03993-5 PMC10827894

[16] Gulcu B, Ozturk E (2022) Endoscopic pilonidal sinus treatment vs. laser-assisted endoscopic pilonidal sinus treatment: short-term results from a retrospective case-matched study. Tech Coloproctol 26(4):271–277 35025023 10.1007/s10151-021-02568-8

[17] Taşkin AK, Özçetin B (2023) Comparison of the effectiveness of laser and crystallized phenol in the treatment of sacrococcygeal pilonidal sinus. Cir Cir 91(3):297–303 37440707 10.24875/CIRU.22000461

[18] Bi S, Sun K, Chen S, Gu J (2020) Surgical procedures in the pilonidal sinus disease: a systematic review and network meta-analysis. Sci Rep 10(1):13720 32792519 10.1038/s41598-020-70641-7 PMC7426950

[19] Basso L, Pietroletti R, Micarelli A, Bicaj A, Costi U, Crocetti D et al (2022) The impact of experience on recurrence rates after biopsy punch excision for pilonidal disease. Colorectal Dis 24(8):984–991 35344244 10.1111/codi.16126 PMC9541250

[20] Ram E, Bachar GN, Goldes Y, Joubran S, Rath-Wolfson L (2018) Modified Doppler-guided laser procedure for the treatment of second- and third-degree hemorrhoids. Laser Ther 27(2):137–142 30087534 10.5978/islsm.18-OR-14 PMC6062676

[21] Sharma D, Pratap A, Ghosh A, Shukla VK (2009) Malignant transformation of a pilonidal sinus. Surgery 145(2):243–244 19167981 10.1016/j.surg.2007.08.018

[22] Sluckin TC, Hazen SJA, Smeenk RM, Schouten R (2022) Sinus laser-assisted closure (SiLaC^®^) for pilonidal disease: results of a multicentre cohort study. Tech Coloproctol 26(2):135–141 34993686 10.1007/s10151-021-02550-4

[23] Iesalnieks I, Ommer A (2019) The management of pilonidal sinus. Dtsch Arztebl Int 116(1–2):12–21 30782310 10.3238/arztebl.2019.0012 PMC6384517

[24] Doll D, Petersen S, Andreae OA, Matner H, Albrecht H, Brügger LE et al (2022) Pit picking vs. Limberg flap vs. primary open method to treat pilonidal sinus disease - A cohort of 327 consecutive patients. Innov Surg Sci 7(1):23–29 35974777 10.1515/iss-2021-0041 PMC9352183

[25] von Oetzmann C, Gödeke J (2021) Pilonidal sinus disease on the Rise: a one-third incidence increase in inpatients in 13 years with substantial regional variation in Germany. Int J Colorectal Dis 36(10):2135–2145 33993341 10.1007/s00384-021-03944-4 PMC8426302

